# Case Report: HHS-spectrum distal palmar arterial thrombosis in a volleyball athlete with provocative-position thoracic outlet narrowing

**DOI:** 10.3389/fcvm.2026.1798334

**Published:** 2026-04-10

**Authors:** Yuwei Wang, Leina Sun, Xiaolong Wang, Hongxu Liu, Longjiang Fang, Fangcheng Su, Sheng-Guang Li, Jingjing Ma

**Affiliations:** 1 Department of Emergency Internal Medicine, Weifang People’s Hospital, Weifang, Shandong, China; 2 School of Traditional Chinese Medicine, Beijing University of Chinese Medicine, Beijing, China; 3 Department of Radiology, Weifang People’s Hospital, Weifang, Shandong, China; 4 Department of Rheumatology and Immunology, Peking University International Hospital, Beijing, China; 5 Department of Rheumatology, Weifang People’s Hospital, Weifang, Shandong, China

**Keywords:** arterial thoracic outlet compression, digital ischemia, hypothenar hammer syndrome, Raynaud phenomenon, subclavian artery compression

## Abstract

**Background:**

Thoracic outlet syndrome (TOS) is a rare condition (∼1–3 per 100,000) caused by neurovascular compression at the thoracic outlet. Arterial involvement is uncommon and may result from chronic or dynamic subclavian artery compression leading to endothelial injury, thrombosis, and distal ischemia. Hypothenar hammer syndrome (HHS) is an uncommon cause of hand ischemia due to repetitive trauma to the ulnar artery and palmar circulation and has been reported in athletes engaged in repetitive high-impact hand activities (e.g., baseball, karate, golf, volleyball).

**Case presentation:**

We report a 16-year-old male volleyball player with a one-month history of cold, pale fingers (Raynaud phenomenon), most pronounced in the right middle finger, accompanied by right upper limb numbness and pain. Initial vascular ultrasound was unremarkable and autoimmune investigations were negative. CTA acquired in an overhead/provocative position demonstrated focal narrowing of the right subclavian artery at the costoclavicular space, compatible with arterial thoracic outlet compression (aTOS-spectrum). Targeted duplex ultrasonography of the hand demonstrated palmar arterial wall irregularity/thickening with intraluminal thrombus and digital ischemia, consistent with distal palmar arterial thrombosis in the HHS spectrum. Provocative maneuvers (Adson's and Roos tests) were positive. The patient underwent ultrasound-guided intra-arterial thrombolysis of the ulnar artery with urokinase, along with anticoagulation and vasodilator therapy (papaverine and prostaglandin E1) and shoulder rehabilitation; symptoms improved mildly on follow-up.

**Conclusion:**

Unilateral Raynaud-like digital ischemia in young athletes may reflect distal palmar arterial thrombosis in the HHS spectrum; provocative-position thoracic outlet compression should be interpreted cautiously, and management should prioritize reperfusion and surveillance.

## Introduction

Thoracic outlet syndrome (TOS) encompasses a spectrum of neurovascular compressive disorders of the brachial plexus, subclavian artery, or subclavian vein as they traverse the thoracic outlet (1). Over 95% of TOS cases are neurogenic, while venous and arterial TOS account for only 3%–5% and ∼1% respectively (2, 3). Arterial TOS (aTOS) typically arises from chronic compression of the subclavian artery (often by a cervical rib, anomalous band, or hypertrophied musculature in athletes), leading to endothelial damage, aneurysmal degeneration, thrombosis, and distal embolization (4). Patients with aTOS may present with limb claudication, hand ischemia, and signs of distal emboli; notably, unilateral Raynaud-type episodes of pallor and cyanosis in the fingers can be a key manifestation (5). However, Raynaud's phenomenon is infrequently caused by TOS (estimated in only 3%–5% of TOS cases), and its presence often prompts evaluation for alternative etiologies (3).

Hypothenar hammer syndrome (HHS) is another rare vascular condition, resulting from injury to the ulnar artery at the level of the hamate from repetitive blunt trauma or a single extreme impact (6). The ulnar artery in Guyon's canal is particularly vulnerable as it lies over the hook of the hamate; repeated pounding (as if using the hand as a hammer) can cause intimal injury, thrombosis or aneurysm of the ulnar artery, and subsequent digital artery embolization (7). HHS typically occurs in males (9:1 male predominance) around the 4th to 5th decade of life and has classically been described in workers using vibrating tools (e.g., jackhammer operators, mechanics) or in individuals who sustain repetitive palm impacts. Notably, various sports have been implicated as risk factors, including baseball (catchers), golf, handball, karate, mountain biking, and even volleyball (8, 9). Patients with HHS often report cold-sensitive digits, hand pain, and signs mimicking Raynaud phenomenon in the ulnar-distribution fingers (ring and small finger), while the thumb is characteristically spared.

We report a 16-year-old competitive volleyball player presenting with refractory Raynaud-like digital ischemia who was found to have arterial thoracic outlet compression (aTOS-spectrum) with distal palmar arterial thrombosis consistent with the HHS spectrum. This case underscores the importance of considering uncommon mechanical causes of digital ischemia in young athletes and highlights a management strategy that prioritizes reperfusion and surveillance, reserving decompression for recurrence or progression.

## Case presentation

A previously healthy 16-year-old right-handed male volleyball player presented with a one-month history of paroxysmal coldness and pallor affecting the fingertips of both hands, more pronounced on the right. He reported that the distal phalanx of the right third finger would blanch and at times turn violaceous, accompanied by numbness and aching pain involving the right upper limb. There was no antecedent major trauma. He denied rashes, alopecia, sicca symptoms, photosensitivity, fever, cough, chest pain, dyspnea, abdominal pain, diarrhea, or inflammatory joint symptoms. On March 10, 2025, he was first evaluated in vascular surgery. Duplex ultrasonography of the right upper-extremity arteries and deep veins showed no hemodynamically significant abnormality or thrombus. Empiric vasodilator therapy (including beraprost sodium) was initiated without improvement. To clarify the etiology of his Raynaud-like episodes, he was admitted to rheumatology with a working diagnosis of Raynaud phenomenon.

**Figure 1:**
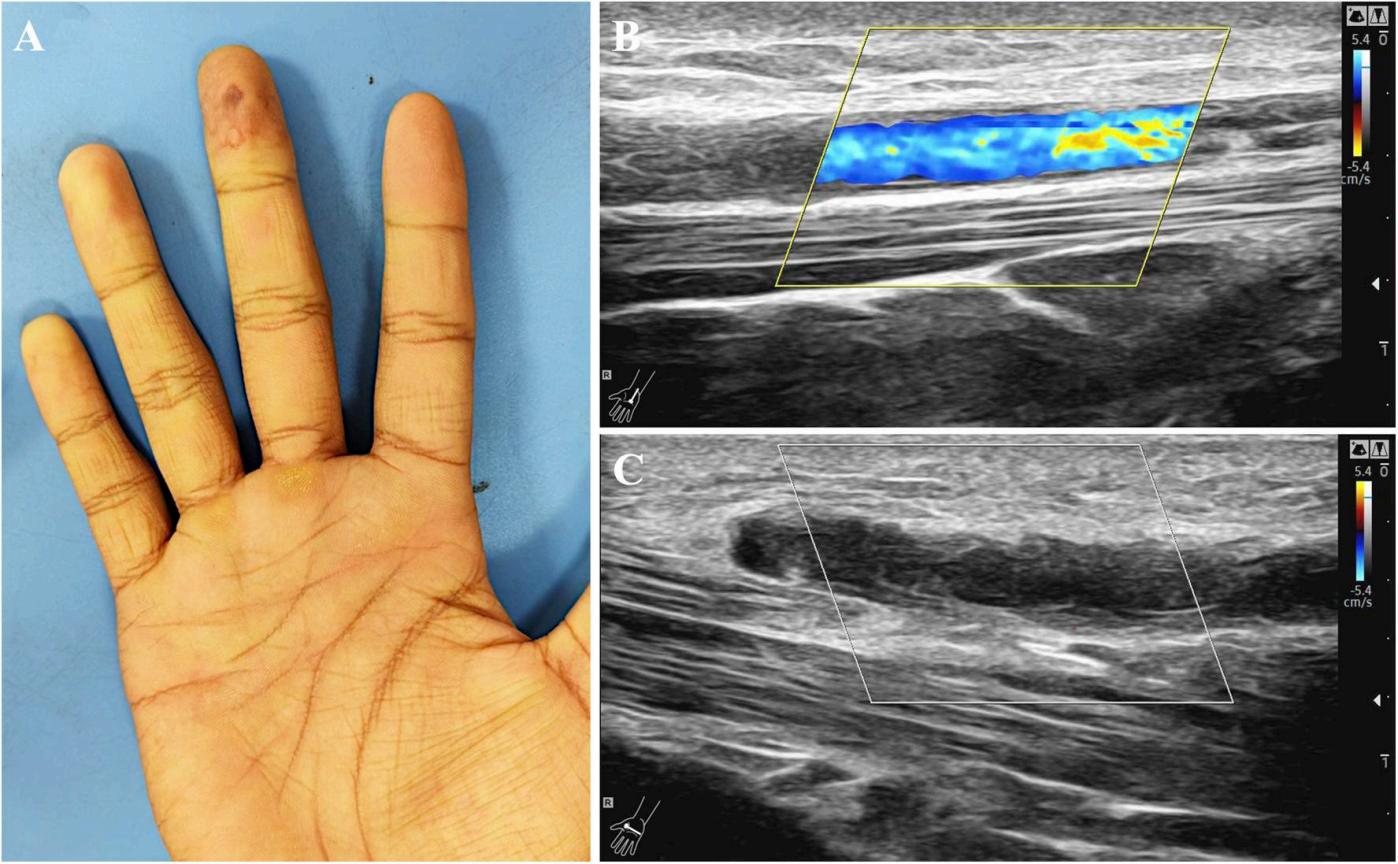
Clinical and duplex ultrasonographic evidence of distal palmar arterial thrombosis (right hand). **(A)** Right-hand photograph showing pallor and violaceous discoloration of the right middle fingertip, consistent with digital ischemia/Raynaud-like change; a yellowish callus over the hypothenar area suggests repetitive impact. **(B)** Color Doppler ultrasound (long-axis view) of the palmar artery demonstrating heterogeneous residual flow with focal flow disturbance. **(C)** Gray-scale ultrasound (long-axis view) demonstrating irregular arterial wall thickening and intraluminal echogenic material consistent with thrombus, supporting distal palmar/ulnar arterial thrombosis with digital ischemia.

On admission, both hands were cool to the touch, with the right markedly colder than the left. The distal right third finger was cyanotic with a sharply demarcated discoloration and was tender to pressure (Figure 1A). There was no cutaneous thickening or sclerosis, no rash, and no joint tenderness. Systemic examination was otherwise unremarkable.

A comprehensive laboratory evaluation for secondary causes of digital ischemia was performed. Complete blood and urine counts were broadly within reference ranges. Autoimmune serologies—including antinuclear antibodies (ANA) and antineutrophil cytoplasmic antibodies (ANCA)—were negative. Serum immunoglobulins and complement levels were unremarkable. Inflammatory markers, including erythrocyte sedimentation rate (ESR) and C-reactive protein (CRP), were normal. These results provided no evidence of connective tissue disease, vasculitis, or systemic inflammation.

**Figure 2:**
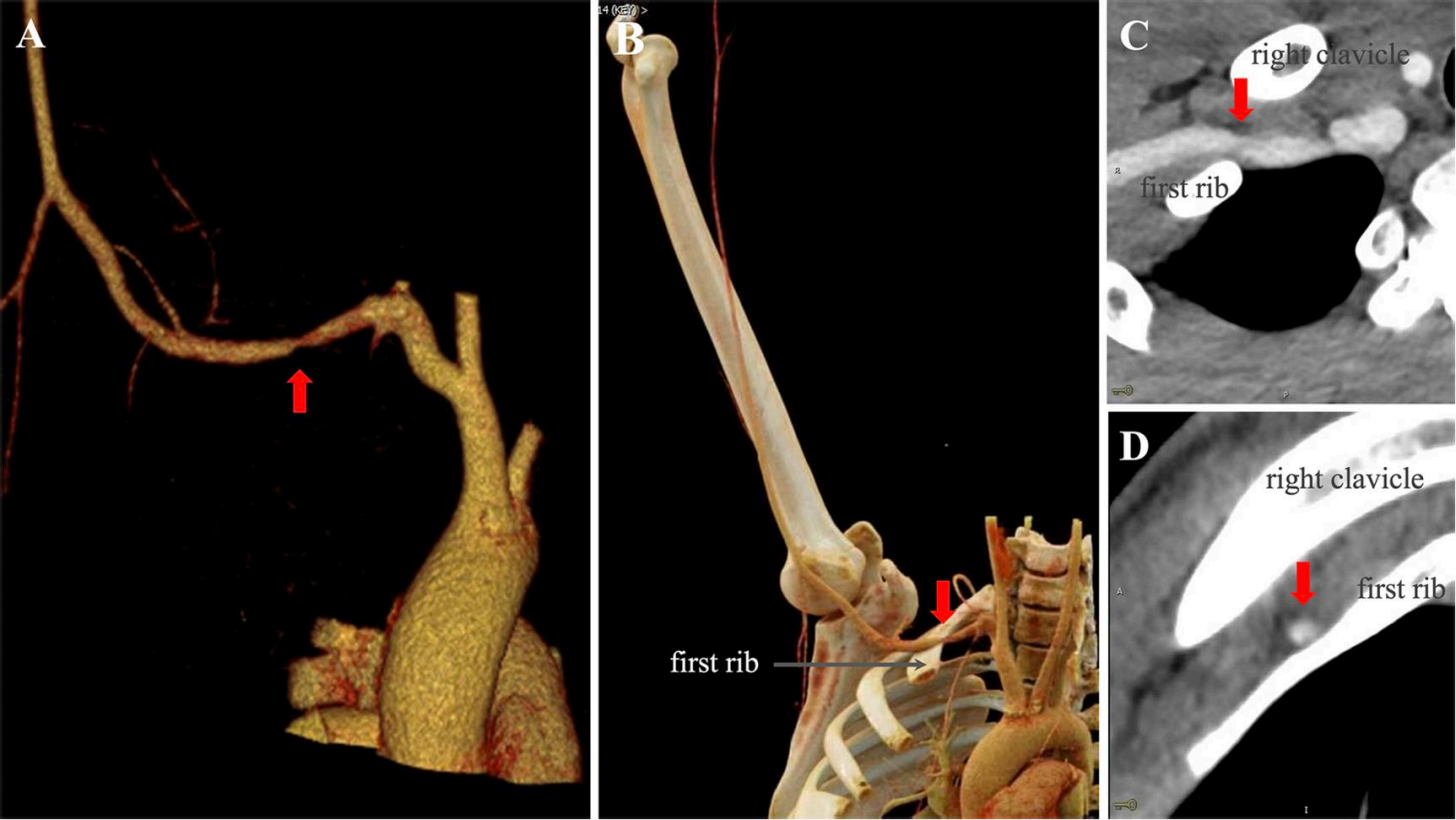
CTA evidence of right subclavian artery compression at the thoracic outlet (overhead/provocative position). **(A)** Three-dimensional CTA rendering demonstrating focal narrowing of the right subclavian artery (arrow). **(B)** Three-dimensional CTA with osseous reconstruction showing the right subclavian artery coursing along the superior margin of the first rib with segmental caliber reduction (arrow). **(C)** Axial CTA image showing luminal narrowing of the right subclavian artery adjacent to the first rib (arrow). **(D)** Sagittal CTA image showing the right subclavian artery traversing the costoclavicular space between the clavicle and first rib with reduced luminal caliber (arrow).

Vascular and musculoskeletal imaging proceeded along a defined timeline. The initial study (March 10, 2025) reaffirmed the vascular surgery assessment: an upper-extremity arterial duplex performed with the arm in a neutral position reported no hemodynamically significant abnormality, and venous duplex revealed no evidence of deep venous thrombosis. On that neutral-position examination, no fixed subclavian arterial stenosis, aneurysm, or mural thrombus was reported. On March 18, contrast-enhanced CT angiography (CTA) of the right upper limb was acquired in an overhead/provocative position and demonstrated focal segmental narrowing of the right subclavian artery at the costoclavicular space (Figures 2A–D). Neutral-position CTA was not available, and no aneurysm or post-stenotic dilatation was identified on the available CTA images. There was no report of an overt cervical rib on this acquisition. On March 19, MRI of the right shoulder identified a small joint effusion and focal soft-tissue edema inferior to the teres minor, without a discrete mass. On the same date, targeted ultrasonography of the right wrist and palm disclosed distal vascular wall changes and downstream flow disturbance: color Doppler (long-axis view) of the palmar artery showed heterogeneous residual flow with focal flow disturbance (Figure 1B), and gray-scale (long-axis view) demonstrated irregular wall thickening with intraluminal echogenic material consistent with thrombus (Figure 1C). Doppler waveforms in the digital arteries supplying the middle finger were diminished, consistent with ischemic compromise. Based on this pattern and the clinical context, the sonographer recommended correlation with repetitive palmar impact to assess for hypothenar hammer syndrome (HHS-spectrum/HHS-like distal palmar arterial thrombosis).

A subsequent focused study (March 21) again documented thickening of the right ulnar arterial wall with roughened palmar arterial contours and thrombus, alongside ischemic changes in the digital circulation of the middle finger. Given the imaging pattern and the clinical context, correlation with repetitive palmar impact was recommended to assess for HHS-spectrum disease.

Given the overhead-position CTA finding and symptom reproduction with positional maneuvers, orthopedic consultation was obtained. Adson's test and the Roos elevated arm stress test reproduced symptoms and were interpreted as compatible with thoracic outlet involvement, whereas Tinel's sign was negative. However, these provocative maneuvers are not specific, and a dedicated dynamic thoracic outlet duplex protocol (neutral vs. provocative positions with standardized subclavian velocity assessment) was not performed; therefore, the thoracic outlet finding was considered supportive but not definitive.

At this juncture, the clinical and duplex findings most strongly supported distal palmar/ulnar arterial thrombosis in the HHS spectrum presenting as unilateral Raynaud-like digital ischemia. The focal right subclavian narrowing on overhead/provocative CTA was interpreted as possible arterial thoracic outlet compression; however, because neutral-position CTA and dedicated dynamic thoracic outlet duplex were not available, a causal relationship between the thoracic outlet finding and the distal palmar thrombosis could not be established.

Therapy proceeded along two parallel tracks: restoration of distal perfusion and mitigation of ongoing thrombotic risk, combined with targeted rehabilitation to address shoulder girdle mechanics. On March 23, under real-time ultrasound guidance, the right ulnar artery was percutaneously accessed in the distal forearm (transulnar access). Intra-arterial thrombolysis was performed by instilling urokinase (total 250,000 IU diluted in 10 mL) into the ulnar–palmar arterial segment harboring thrombus. The procedure was performed without fluoroscopic angiography/DSA; therefore, angiographic images were not available, and transfemoral or transbrachial access was not used. Given the distal localization of the thrombus and clear sonographic visualization of the involved segment, an ultrasound-guided local intra-arterial approach was deemed appropriate. Concomitantly, systemic anticoagulation with heparin was initiated. Vasodilator therapy included papaverine to reduce arterial spasm and alprostadil (prostaglandin E1) to augment microcirculatory flow. In parallel, a structured shoulder rehabilitation program was prescribed, emphasizing postural correction and shoulder girdle mobility with the intent to minimize dynamic vascular compression during overhead activity.

Across the admission, no clinical or serologic features emerged to suggest an alternative systemic etiology. The patient's cutaneous examination remained free of scleroderma stigmata, no inflammatory arthritis developed, and inflammatory indices stayed within normal limits. Serial bedside assessments continued to note a temperature gradient between hands with right-sided predominance early in the course; the right third fingertip remained the most symptomatic site. No digital ulceration or tissue loss was recorded during the hospitalization. The patient tolerated endovascular thrombolysis and the initiation of anticoagulation without adverse events.

The discharge plan included continuation of antithrombotic therapy as per vascular protocol, a vasodilator regimen to be tapered according to symptom control, strict thermal protection of the hands, and activity modification to reduce repetitive impact to the hypothenar region. He was counseled to avoid provocative overhead positions and high-impact palm strikes inherent to his sport during convalescence and while completing rehabilitation. Outpatients follow-up with vascular surgery and rehabilitation medicine was arranged. At outpatient follow-up, the patient reported mild symptomatic improvement.

In sum, a one-month evolution of Raynaud-type digital ischemia in a teenage overhead athlete culminated in the identification of localized vascular pathology at two levels of the right upper limb circulation, confirmation by targeted imaging and provocative testing, and prompt initiation of endovascular, pharmacologic, and rehabilitative therapies tailored to the composite diagnosis established during hospitalization.

## Discussion

This case highlights that juvenile, unilateral Raynaud-like episodes can reflect structural vascular pathology rather than primary vasospasm. In young overhead athletes, distal palmar/ulnar arterial thrombosis in the hypothenar hammer syndrome (HHS) spectrum can present with painful, asymmetric color change and threatened digital ischemia that mimics Raynaud phenomenon. Apparent thoracic outlet arterial compression identified only on overhead/provocative CTA may coexist but should be interpreted cautiously and not assumed causal without corroborating dynamic hemodynamic testing or evidence of fixed subclavian arterial injury. Literature from sports medicine and vascular surgery underscores that mechanically mediated arterial lesions in athletes can masquerade as “Raynaud's” (10).

aTOS is the least frequent TOS subtype (≈1%–2% of cases), but it is disproportionately represented in athletes who repetitively place the shoulder in abduction-external rotation. Chronic compression of the subclavian artery at the scalene triangle or costoclavicular space causes intimal injury, segmental stenosis, and sometimes poststenotic aneurysm with mural thrombus; embolization to the hand can follow (4). Prior reports emphasize that unilateral digital ischemia in the dominant arm is a red flag for TOS or axillary/subclavian arterial lesions and merits urgent vascular imaging with dynamic maneuvers (10). Return-to-play data after definitive surgery are favorable in elite athletes, strengthening the case for early recognition.

HHS arises from repetitive blunt impact of the hypothenar eminence against the hook of the hamate, where the ulnar artery and superficial palmar arch are vulnerable. Pathology ranges from intimal tears and non-occlusive wall thickening to thrombosis or pseudoaneurysm; embolization then seeds the ulnar-distribution digits. Classic angiography may show the “corkscrew” ulnar artery. Although historically linked to male manual workers, HHS is also recognized in athletes (including volleyball), reflecting sport-specific palmar impacts (11, 12). Diagnostic pathways place duplex ultrasound up front and reserve angiography for confirmation or intervention planning (13).

The present case demonstrated duplex-documented palmar arterial wall irregularity/thickening with intraluminal thrombus and diminished digital flow, supporting HHS-spectrum distal palmar thrombosis as the most parsimonious explanation for the threatened digit. Overhead-position CTA additionally showed focal right subclavian narrowing at the costoclavicular space (Figures 2A–D); however, because neutral-position CTA and dedicated dynamic thoracic outlet duplex were not available, we cannot determine whether this represents clinically significant thoracic outlet compression or physiologic positional narrowing. Accordingly, we present the thoracic outlet finding as a potential concomitant factor or incidental imaging observation and emphasize that provocative-position CTA narrowing alone does not establish an embolic source for distal palmar thrombosis (14, 15). Practical algorithms advise that when duplex suggests HHS-spectrum disease, clinicians may still consider CTA/MRA to assess for a proximal arterial lesion, while interpreting provocative-only compression with caution (6).

Recognizing secondary (“structural”) Raynaud. Features that should trigger evaluation beyond primary Raynaud include asymmetry, painful attacks, objective ischemia/ulceration, abnormal pulses, and poor response to vasodilators. Contemporary reviews and guidelines emphasize nailfold capillaroscopy and autoimmune serology to exclude connective-tissue diseases, while keeping a parallel differential that includes embolic, traumatic, and compressive vascular etiologies—especially in young males or athletes (16). In this patient, negative serologies and normal inflammatory markers refocused attention on mechanical disease.

For suspected aTOS, static imaging can miss positional compromise. Dynamic duplex with provocative arm positions and dynamic CT angiography (e.g., ABER positioning) improve sensitivity; lesions exceeding ≈50% dynamic stenosis correlate with symptoms and guide decisions (17). Consensus statements encourage standardized reporting and incorporation of dynamic assessments into TOS work-ups. For HHS, high-resolution duplex identifying ulnar wall irregularity/thrombus often suffices to proceed to therapy; when needed, angiography confirms the diagnosis and delineates the palmar arch (18). In our case, the initial neutral-position upper-extremity arterial duplex was reported as unremarkable; CTA was obtained only in an overhead/provocative position without neutral-position comparison, and a dedicated dynamic subclavian duplex protocol was not performed. Therefore, we cannot quantify the dynamic component or fully exclude physiologic positional narrowing, nor can we establish causality between the thoracic outlet finding and the distal palmar thrombosis.

Therapeutic priorities—salvage the threatened digit, then address the source. The immediate goal in hand-threatening ischemia is reperfusion. In acute or subacute HHS-related thrombosis (days to ≤2 weeks), catheter-directed thrombolysis is an accepted option, with reports of successful local urokinase infusion restoring flow; antithrombotic therapy and vasodilators complement lysis, along with strict avoidance of further palmar trauma (19). Chronic HHS with pseudoaneurysm or organized occlusion often requires surgical ligation or segmental resection with reconstruction, provided the radial system can sustain the hand (8, 20).

In aTOS, definitive management depends on arterial morphology. When aneurysm, wall thrombus, or recurrent embolization is present, most series recommend thoracic outlet decompression (first-rib resection and scalenectomy) combined with arterial repair (aneurysmectomy, patch, or interposition graft) and clearance of distal thrombi (1, 2). In less advanced disease without aneurysm—particularly in very young patients—some advocate a trial of structured rehabilitation to optimize shoulder girdle mechanics while surveilling the artery; emergent thrombolysis is reserved for acute limb-threatening thrombosis (21, 22). The overhead-athlete literature still treats surgery as the standard for embolic or aneurysmal disease, aiming to eliminate the source and facilitate return to sport (23). In this patient, the absence of aneurysm/post-stenotic dilatation on available CTA and mild symptomatic improvement after thrombolysis supported an initial strategy of reperfusion plus rehabilitation with close surveillance, reserving decompression for recurrence, progression, or development of fixed arterial lesions.

Structured therapy emphasizing scapular stabilization, scalene/pectoralis minor flexibility, and technique modification can reduce dynamic compression in overhead athletes. Even when surgery is performed, formal rehabilitation is integral to outcomes and timing of return. Reports in elite throwers show most athletes resume high-level competition within months after definitive management; though extrapolation to adolescents requires caution, these data support an aggressive functional recovery plan. For HHS, padding the hypothenar region and modifying ball-contact techniques reduce reinjury risk (24, 25).

Unilateral digital ischemia in youth invites a wide differential: connective-tissue disease (largely excluded by serology and capillaroscopy), thromboangiitis obliterans (unlikely in non-smokers), vasculitis, cardioembolism, hypercoagulable states, entrapment at other sites (e.g., vascular quadrilateral space), and local trauma. Reviews on digital ischemia emphasize aligning the pattern (distribution, symmetry, triggers) with plausible mechanisms and using imaging to localize the lesion; in our case, the dual-level imaging findings were decisive (26).

## Conclusion

This case underscores that unilateral Raynaud-like digital ischemia in young athletes may reflect distal palmar/ulnar arterial thrombosis in the HHS spectrum. Apparent thoracic outlet arterial compression on provocative imaging should be interpreted cautiously and does not, by itself, establish causality; timely reperfusion and structured vascular surveillance are essential, and thoracic outlet decompression should be reserved for recurrent symptoms or objectively proven fixed subclavian arterial injury.

## Data Availability

The original contributions presented in the study are included in the article/Supplementary Material, further inquiries can be directed to the corresponding authors.
